# Role of PPARs in *Trypanosoma cruzi* Infection: Implications for Chagas Disease Therapy

**DOI:** 10.1155/2012/528435

**Published:** 2012-02-09

**Authors:** Eugenia Hovsepian, Federico Penas, Gerardo A. Mirkin, Nora B. Goren

**Affiliations:** ^1^Centro de Estudios Farmacológicos y Botánicos (CEFYBO, CONICET, UBA), Buenos Aires 1121, Argentina; ^2^Departamento de Microbiología, Parasitología e Inmunología, Facultad de Medicina, Universidad de Buenos Aires, Buenos Aires 1121, Argentina

## Abstract

Chagas disease, which is caused by *Trypanosoma cruzi* (*T. cruzi*), remains a substantial public health concern and an important cause of morbidity and mortality in Latin America. *T. cruzi* infection causes an intense inflammatory response in diverse tissues by triggering local expression of inflammatory mediators, which results in the upregulation of the levels of cytokines and chemokines, and important cardiac alterations in the host, being one of the most characteristic damages of Chagas disease. Therefore, controlling the inflammatory reaction becomes critical for the control of the proliferation of the parasite and of the evolution of Chagas disease. The nuclear receptors known as peroxisome proliferator-activated receptors (PPARs) have emerged as key regulators of lipid metabolism and inflammation. The precise role of PPAR ligands in *T. cruzi* infection or in Chagas disease is poorly understood. This review summarizes our knowledge about *T. cruzi* infection as well as about the activation of PPARs and the potential role of their ligands in the resolution of inflammation, with the aim to address a new pharmacological approach to improve the host health.

## 1. Introduction

Chagas disease is widely distributed throughout Latin America, thus causing a serious public health problem. It is considered the parasitic disease that leads to the greatest economic burden in Latin America due to its prolonged chronicity. Reasonably, public control programs normally focus their resources and strategies on the elimination of insect vectors associated with human habitat and relegate the infected patient. Moreover, migration from rural to urban areas has changed the epidemiology of the disease. While in the 1930's 70% of Latin Americans lived in rural areas, currently about 70% live in urban areas. The infection was primarily rural and became urban transmissible by blood transfusion. In recent decades, the number of donors with positive serology has increased in endemic countries. Thus, there is a need for new strategies to prevent or stop the cardiac consequences of *T. cruzi* infection, with an affordable cost to the health system and patients. The host responds to invasion by the activation of inflammation and induction of innate and specific immunity. The infectious inflammatory myocarditis generated by *T. cruzi* induces an inflammatory response that affects the heart tissue and function. Moreover, the immune system can act with autoimmune reaction with infiltration of macrophages and/or cell-damaging attack. Therefore, the anti-inflammatory actions of peroxisome proliferator-activated receptors (PPARs) *γ* and *α* have received great attention because of the availability of synthetic PPAR activators. PPAR ligands emerge as attractive drug targets for lipid and glucose metabolism as well as for inflammation resolution. The aim of this article is to review the role of PPARs in *T. cruzi *infection and their potential contribution to inflammation resolution.

## 2. Chagas Disease

Chagas disease, also called American trypanosomiasis, is a chronic and systemic parasitic infection caused by the protozoan *Trypanosoma cruzi*. This parasite was discovered in 1909 by the Brazilian physician Carlos Chagas (1879–1934), who described in detail the cycle of transmission and the human clinical manifestations.

This infectious disease is endemic throughout Central and South America and is still recognized by the World Health Organization (WHO) as one of the most important ignored tropical diseases and as a significant public health problem. In recent decades, the increased rate of emigration from Chagas-endemic countries to the United States, Canada, and the European Union has become a new concern for the WHO [1].

*T. cruzi* has several instances of transmission to humans and other susceptible hosts, mainly through contact with the feces of infected blood-feeding insect vectors. However, alternative routes such as blood transfusion, organ transplant, congenital transmission and oral transmission have also been determined [2].

The clinical course of the infection has two phases: an acute and a chronic. The acute phase is characterized by evident parasitemia and parasitism in a wide variety of host cells. This phase can be confused with other infections since symptoms such as fever or hepatomegaly and/or splenomegaly are shared by different infectious diseases. Most of the patients that survive the acute phase remain in a life-long asymptomatic state (indeterminate form) during the chronic phase of infection [3].

During the acute phase of infection, most individuals usually have mild symptoms such as fever, which do not need medical attention, with few or no parasites found in circulation. Symptoms of acute infection may last up to a few weeks or months. Although the infection then remains largely asymptomatic, often for years or even decades, 30% of patients develop chronic Chagas disease [4].

Symptoms of the acute phase resolve spontaneously in about 90% of infected patients even if the infection is not treated with trypanocidal drugs. About 60–70% of these patients will never develop clinically apparent disease. These patients have the indeterminate form of chronic Chagas disease. The remaining 30–40% of patients will develop a form of chronic disease characterized by progression to cardiac disease, gastrointestinal disease, or both, over a period of years to decades [3, 5, 6].

The cardiomyopathy in South and Central America develops manifestations like cardiac arrhythmias, apical aneurysms, congestive heart failure, thromboembolism, and sudden cardiac death in disease-endemic areas [7]. As expected, Chagas disease can be reactivated in patients with HIV/AIDS or subject to chemotherapy [8].

It has been described that Chagas disease is typified by a chronic inflammatory process that causes damage to the myocardium as well as to the conduction system. The pathogenesis may involve several mechanisms, including immunologically mediated tissue damage, cardiac dysfunction, and coronary microvascular disease [9]. There are substantial evidences showing that cardiac tissue, an important target of *T. cruzi,* produces marked amounts of proinflammatory cytokines, chemokines, and enzymes including inducible nitric oxide (NO) synthase (NOS2) and metalloproteinases (MMPs), resulting in inflammation and cardiac remodeling in response to parasite infection [10–12].

## 3. The Peroxisome Proliferator-Activated Receptors (PPARs) Family

PPARs are members of the nuclear receptor superfamily of ligand-activated transcription factors. The PPAR subfamily (NR1C) includes PPAR-*α* (NR1C1), PPAR-*β* (also called PPAR-*δ*) and NUC1, NR1C2, and PPAR-*γ* (NR1C3) [13], each with different ligands, target genes, and biological role. Most of these PPARs share a similar structure, which includes an amino-terminal activation domain (AF-1), a DNA-binding domain, a ligand-binding domain, and a second carboxy-terminal activation domain (AF-2) [14]. In response to ligand binding, these receptors change their conformational structure recruiting coactivators and freeing corepressors. The PPAR family not only regulates metabolic processes but also participates in extrametabolic processes, including direct activation of genes, ligand-independent repression, ligand-dependent repression, and transrepression [15].

**Figure 1:**
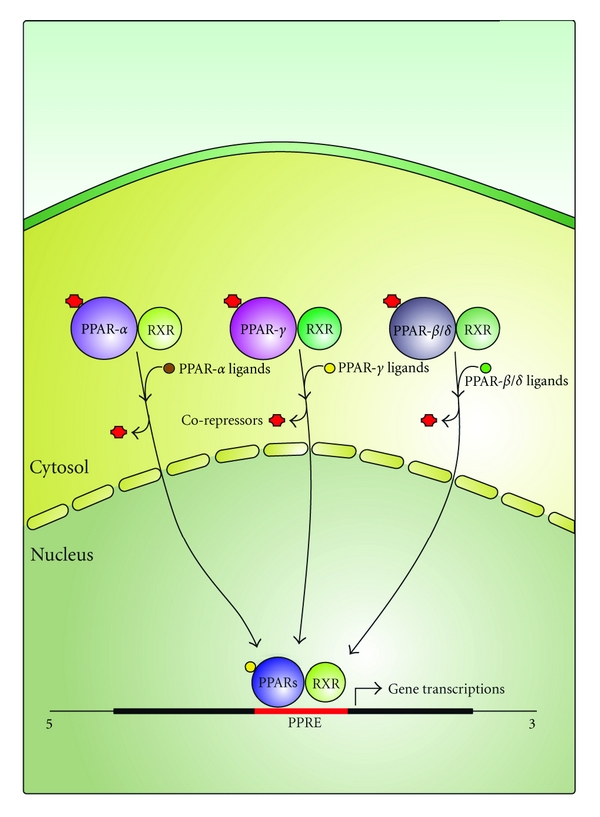
A proposed mechanism of ligand-mediated activation of PPARs. In response to ligand binding, PPARs undergo conformational changes in protein structure. This allows dissociation of corepressor proteins which inhibit transcription and the recruitment of co-activators. PPAR-RXR heterodimers bind to specific recognition sites of DNA, termed peroxisome proliferator activated receptor response elements (PPREs) located in the regulatory region of target genes.

Nuclear receptors can be activated by ligand-dependent or -independent mechanisms. PPARs are activated by xenobiotics as well as by endogenous fatty acids and their metabolites. PPARs activate the transcription of their target genes as heterodimers with retinoid X receptors (RXRs), which are activated by 9-cis retinoic acid [16, 17]. Eicosanoids are some of the endogenous ligands that bind to the PPAR-RXR complex, leading to conformational changes, freeing the co-repressor, and thus binding to the response element of target genes [15] (Figure 1). PPARs have been cloned in several species, including rodents, amphibians, teleosts, cyclostomes, and even humans. The PPAR subtypes (*α*, *β*, and *γ*) are expressed differently according to the tissue but may also be coexpressed in different relative concentrations [18].

### 3.1. PPAR-*α*

PPAR-*α* was identified in the early 1990s on the basis of it being a target of hypolipidemic fibrate drugs and other compounds that induce peroxisome proliferation in rodents [19]. PPAR-*α* is expressed in cells that have active fatty acid oxidation like hepatocytes, cardiomyocytes, enterocytes, smooth muscle cells, and kidney cells and has been implicated in the regulation of cellular energetic processes. It has been shown that PPAR-*α* ligands, such as fibrates, decrease triglyceride levels and reduce the incidence of cardiovascular events and atherosclerosis [20]. The first evidence indicating a potential role for PPARs in the inflammatory response was the demonstration that leukotriene B4, a proinflammatory eicosanoid, binds to PPAR-*α* and induces the transcription of genes involved in *ω*- and *β*-oxidation [21]. It has been described that PPAR-*α* is expressed in human and mouse immune cells, including lymphocytes, macrophages, and dendritic cells, and numerous studies have implicated PPAR-*α* in the negative regulation of inflammatory responses. Different works using PPAR-*α* ligands have shown a reduction in the symptoms of inflammation and disease in several models, including models of allergic airway disease, arthritis, and inflammatory intestine disease [22]. Moreover, the role of PPAR-*α* in the heart has been shown with regards not only to the governing of myocardial energy metabolism and function (using both gain-of-function and loss-of-function murine models) but also to extrametabolic activities such as anti-inflammatory activities (see [23, 24], respectively, for a review). There are many works that have further implicated PPAR-*α* as an important regulator of inflammatory disease. For instance, it has been demonstrated that PPAR-*α* activators inhibit IL-1*β*-induced IL-6 secretion by human aortic smooth muscle cells in a dose-dependent manner [25]. PPAR-*α* activators also negatively regulate IL-1*β*-induced-IL-6 production at the gene expression level by inhibiting NF-*κ*B transcriptional activity [26]. In addition, other mechanisms, including alterations in cytokine-receptor and growth-factor receptor signaling, and the upregulation of the expression of a subunit of the inhibitor of NF-*κ*B (I*κ*B), have been reported (see [24] for a review). 

### 3.2. PPAR-*β*/*δ*

PPAR-*β*, also known as PPAR-*δ*, is expressed ubiquitously and often at higher levels than PPAR-*α* and PPAR-*γ*, suggesting a fundamental role for it in many tissues. PPAR-*δ* was first identified in *Xenopus laevis* [27], and the mouse and human receptors were subsequently cloned on the basis of sequence similarity with PPAR-*α* [17]. PPAR-*δ* target genes in metabolic tissues are broadly involved in fatty acid metabolism, mitochondrial respiration, and thermogenesis. An in vitro study in endothelial cells has indicated that PPAR-*β*/*δ* ligands inhibit TNF*α*-induced upregulation of the expression of VCAM-1, MCP-1, and NF-*κ*B translocation [28]. The role of PPAR-*δ* in the modulation of inflammation is poorly understood. It has been proposed that, in macrophages, PPAR-*β*/*δ* also controls inflammation by its association and disassociation with the transcriptional repressor BCL-6. It has also been described that the loss of hematopoietic PPAR-*δ* expression protects against atherosclerosis, being the proatherogenic effects of PPAR-*δ* due in part to the influence of PPAR-*δ* on the basal expression of inflammatory mediators in the arterial wall. It has also been found that PPAR-*δ* ^−/−^ bone-marrow-derived macrophages show reduced expression of CCL2, matrix metalloproteinase 9 (MMP9), and IL-1*β* and that the ligand binding to PPAR-*δ* releases BCL-6, resulting in the repression of inflammatory gene expression [29].

### 3.3. PPAR-*γ*

PPAR-*γ* is the most studied member of the PPAR family. Two distinct isoforms of PPAR-*γ* (PPAR-*γ*1 and PPAR-*γ*2), which are derived from the same gene but arise by differential transcription start sites and alternative splicing, have been described [30]. This receptor has been cloned from a number of species, including mice, hamsters, frogs, pigs, monkeys, and humans [17, 27, 31]. PPAR-*γ* has a prominent expression in brown and white adipose tissue, the colon, differentiated myeloid cells and the placenta [32]. Brown and white adipocyte tissues are major sites for PPAR-*γ* expression. In 1995, Greene et al. identified two transcripts corresponding to a full-length mRNA and a short form devoid of functional domains [31]. More recent studies have demonstrated that the full-length PPAR-*γ* is indeed expressed in activated T and B cells and monocytes/macrophages [33–35].

The main physiological function of PPARs is the modulation of the expression of specific target genes [36]. PPAR-*γ* is critical for the differentiation of preadipocytes to adipocytes and also participates in glucose metabolism homeostasis [37, 38]. PPAR-*γ* can be activated by several physiological ligands, such as docosahexaenoic acid, linoleic acid, and some synthetic ones like antidiabetic glitazones, which are used as insulin sensitizers. Other ligands include oxidized LDL, azoyle PAF, and eicosanoids, such as 5,8,11,14-eicosatetraenoic acid and the prostanoids PGA_1_, PGA_2_, PGD_2_, and the dehydration products of the PGJ series of cyclopentanones, for example, 15-deoxy-Δ12,14-PGJ2 (15dPGJ2) [39]. In particular, the last one is recognized as an endogenous ligand for the intranuclear receptor PPAR-*γ* being responsible for many anti-inflammatory functions (see [40] for a review). However, previous studies have reported that PPARs inhibit inflammatory gene expression by several mechanisms, including direct interactions with AP1 and NF-*κ*B [41–43], nuclear cytoplasmic redistribution of the p65 subunit of NF-*κ*B [44], modulation of p38 mitogen-activated protein kinase activity [45], competition for limiting pools of coactivators [46], and interactions with transcriptional co-repressors [29] (see [47] for a review).

PPAR-*γ* has been reported to regulate inflammatory responses, both in vivo and in vitro, being involved in the regulation of macrophages and endothelial cells, both crucial to the inflammatory response. The presence of PPAR-*γ* in macrophages was first described in studies using human atheromas [48–51]. A role for PPAR-*γ* in T-lymphocyte regulation has also been described [52]. Furthermore, PPAR-*γ* is involved in the differentiation and activation of monocytes and in the regulation of their inflammatory responses. Previous works have suggested that different stimuli increase monocytes/macrophages PPAR-*γ* expression [53, 54]. Anti-inflammatory effects of macrophage PPAR-*γ* activation have been demonstrated in a number of studies [55, 56].

The roles of 15dPGJ2 and PPAR-*γ* in the regulation of human autoimmune diseases or in animal models of autoimmunity have been studied by several groups.

15dPGJ2 and other PPAR-*γ* ligands inhibit inflammation in models of arthritis [57–59], ischemia-reperfusion injury [60], inflammatory bowel disease (IBD) [61–63], Alzheimer's disease [64–66], and lupus nephritis [67, 68]. 

## 4. Can PPAR Promote or Prevent the Inflammatory Response after *T. cruzi* Infection?

Chagas disease is caused by infection with the protozoan kinetoplastid parasite *Trypanosoma cruzi*. Acute *T. cruzi* infection is accompanied by an intense inflammatory reaction in many tissues, being usually asymptomatic. When symptoms occur, they include prolonged fever, enlargement of the liver, spleen, and lymph nodes, subcutaneous edema (chagoma) or edema of the ocular mucous membranes (Romaña's sign).

There are substantial evidences showing that the cardiac tissue is an important target of *T. cruzi* infection. Controlling the inflammatory reaction is critical for the control of the parasite proliferation in all the tissues, especially in the heart since it may progress to fibrosis and remodeling, resulting in a dilated cardiomyopathy accompanied by myocardial dysfunction [69]. In the context of inflammatory response, in a review of parasitic infections, Chan et al. (2010) argue that PPAR activation might favor the establishment of a chronic parasitic infection, making symbiotic survival between the host and parasite more probable [70]. In this sense, the adipose tissue has been identified as one of the main sites of inflammation during Chagas disease progression when cultured adipocytes are infected with the Tulahuen strain of *T. cruzi, *demonstrating an increase in the expression of proinflammatory mediators [71]. Fnu Nagajyothi et al. (2008) described an infection-associated decrease in adiponectin and PPAR-*γ* in infected adipocytes. They also showed that PPAR-*γ* is highly expressed in adipose tissue and that, together with the adipokine adiponectin, represses the inflammatory process although the mechanism by which adiponectin exerts an anti-inflammatory effect is unclear [71]. However, it is clear that a reduction in the level of adiponectin is associated with an increase in inflammation and that there is an inverse relationship between PPAR-*γ* and inflammation [71]. Another group of researchers has recently described in a mouse model of infection with the Colombian strain of *T. cruzi *(MHOM/CO/00/Colombian) that the treatment with 15dPGJ2 reduces the inflammatory infiltrate in the skeletal muscle at the site of infection and decreases the number of lymphocytes and neutrophils in the blood. These researchers also found that 15dPGJ2 also decreases the relative volume density of parasitic nests in cardiac muscle [72]. Many works have described that PPAR-*α* and PPAR-*γ* agonists play an important role in regulating inflammation in different models in vivo and in vitro. Recently, the role of rosiglitazone, a PPAR-*γ* synthetic agonist, in the modulation of the innate immune response has been demonstrated in an experimental cerebral malaria model (Reviewed in [70]). Also, it has been reported that rosiglitazone together with antischistosomal drugs improves the symptoms of liver fibrosis induced by *Schistosoma japonicum *in mice [70]. Besides, by linking metabolism and immunity, Gallardo-Soler et al. proposed that PPAR activity induces macrophage activation toward a more Th2 immune phenotype in a model of *Leishmania major* infection. These authors showed that PPAR-*γ* and PPAR-*δ* ligands promote intracellular amastigote growth in infected macrophages, and that this effect is dependent on both PPAR expression and arginase activity, suggesting that Arginase I is a key marker of the alternative program triggered by PPAR in macrophages [73].

**Figure 2:**
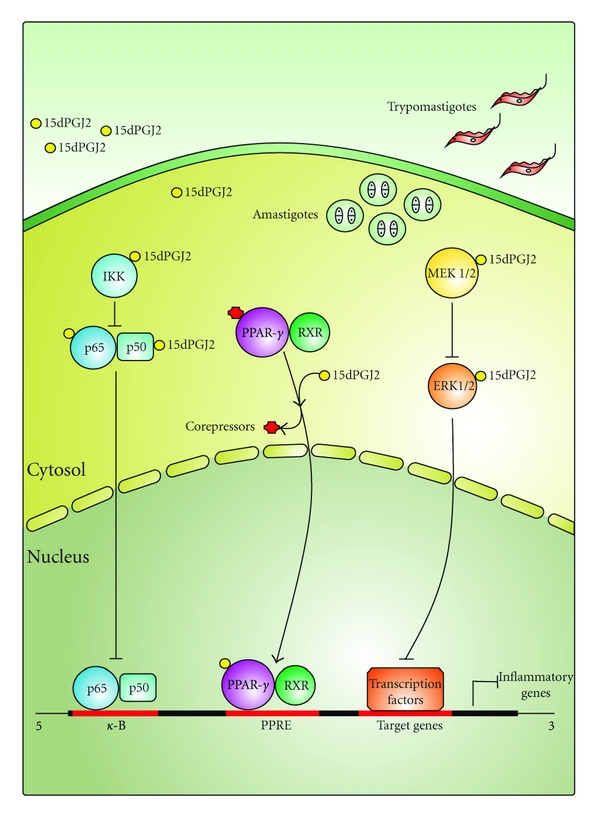
Anti-inflammatory actions of 15dPGJ2 in *T. cruzi*-infected cardiomyocytes. 15dPGJ2 can exert its effects by binding to PPAR-*γ* or through interaction with intracellular targets like NF-*κ*B-signaling pathway and Erk MAP kinase cascade. By PPAR-*γ*-dependent mechanisms, the 15dPGJ2-PPAR-*γ* complex forms a heterodimer with nuclear retinoid X receptor (RXR) to recognize PPAR-response elements (PPREs) in the promoter region of the target genes thereby stimulating their transcription. In the cytosol, 15dPGJ2 can also bind specific residues in IKK, p50, or p65 of the NF-*κ*B-signaling pathway, or MEK1/2 and Erk1/2 in the MAP kinase pathway leading to functional inactivation of inflammatory target genes. The consequent inhibition of inflammatory factors/mediators by 15dPGJ2 promotes an increase in the number of intracellular parasites.

*Trypanosoma cruzi* infection causes an intense inflammatory response in diverse tissues, including the heart. The inflammatory reaction is critical for the control of the parasites' proliferation and evolution of Chagas disease. 15dPGJ2 can repress the inflammatory response in many experimental models. However, the precise role of PPAR-*γ* ligands in *T. cruzi* infection is poorly understood. Hovsepian et al. (2011) have recently reported the first evidence that 15dPGJ2 treatment increases the number of intracellular parasites and inhibits the expression and activity of different inflammatory enzymes such as inducible nitric oxide synthase (NOS2), matrix metalloproteinases 2 and 9 (MMP-2, MMP-9), as well as proinflammatory cytokine (TNF-a and IL-6) mRNA expression in neonatal mouse cardiomyocytes after *T. cruzi* infection [12] (Figure 2). They also observed that transfection of cardiomyocytes with small interfering RNA (siRNA) induces silencing of PPAR-*γ*, impairing the effects of 15dPGJ2 on the modulation of proinflammatory enzymes. In addition, they found that transfection restores the ability of these cells to control the intracellular growth of *T. cruzi *[12]. Like other nuclear receptors, PPARs are phosphoproteins and their transcriptional activity is affected by crosstalk with kinases and phosphatases. In addition, 15dPGJ2 can act in a ligand-dependent or -independent manner through mitogen-activated protein kinase (MAPK) and nuclear factor-*κ*B (NF-*κ*B) [74]. Hovsepian et al. (2011) demonstrated in *T. cruzi*-infected neonatal cardiac cells that PPAR-*γ*-independent pathways are involved, since 15dPGJ2 also exerts its effect through extracellular signal-regulated kinases-mitogen-activated protein kinase (Erk-MAPK) and NF-*κ*B. The use of specific pharmacological inhibitors confirmed these findings [12] (Figure 2). Our group has recently found evidence about the role of 15dPGJ2 in the regulation of inflammation parameters in a mouse-experimental model of *T. cruzi* infection, confirming all the results assayed in neonatal cardiomyocytes (data not shown, sent manuscript). In this manuscript, we report evidences that 15dPGJ2 treatment inhibits TNF*α* and IL-6 mRNA levels as well as the expression and activity of inflammatory enzymes like NOS2 and MMP-2. We found that 15dPGJ2 participates in both parasitemia and amastigote nest regulation in hearts of infected mice and that it does not modify the mortality rate in acute infection. In the presence of GW9662, a specific PPAR-*γ* antagonist, 15dPGJ2 partially inhibited NOS2 expression and MMP-2 activity, denoting the participation of some other signaling pathway. We also found that NF-*κ*B was activated by means of p65 nuclear translocation in hearts of infected mice and inhibited after 15dPGJ2 treatment. These results highlight that both PPAR-*γ* and NF-*κ*B are implicated in the inhibitory effects of 15dPGJ2 on the inflammatory response of the heart in an acute model of *T. cruzi* infection (data not shown).

## 5. Perspectives

To date, the accurate role of PPAR ligands in *T. cruzi* infection remains essentially unexplored. In this sense, some authors argue that the expression of PPAR-*γ* decreases after *T. cruzi* infection, while others argue that this expression increases. However, in general, most research groups highlight the role of PPARs in resolving inflammation and collaborating with tissue repair in some cases. Therefore, we hope that PPARs can potentially contribute to address a new pharmacological approach to improve the host health.
